# Strengthening the national health information system through a capacity-building and mentorship partnership (CBMP) programme: a health system and university partnership initiative in Ethiopia

**DOI:** 10.1186/s12961-021-00787-x

**Published:** 2021-12-09

**Authors:** Binyam Tilahun, Kassahun D. Gashu, Zeleke A. Mekonnen, Berhanu F. Endehabtu, Moges Asressie, Amare Minyihun, Adane Mamuye, Asmamaw Atnafu, Wondimu Ayele, Keneni Gutema, Admas Abera, Mulumebet Abera, Teklit Gebretsadik, Biruk Abate, Mesoud Mohammed, Netsanet Animut, Hiwot Belay, Hibret Alemu, Wubishet Denboba, Abebaw Gebeyehu, Naod Wondirad, Lia Tadesse

**Affiliations:** 1 CBMP Program, University of Gondar, Gondar, Ethiopia; 2 CBMP Program, Addis Ababa University, Addis Ababa, Ethiopia; 3 CBMP Program, Hawassa University, Hawasa, Ethiopia; 4 CBMP Program, Haromaya University, Harar, Ethiopia; 5 CBMP Program, Jimma University, Jimma, Ethiopia; 6 CBMP Program, Mekele University, Mekele, Ethiopia; 7 Policy and Planning Directorate, Ministry of Health, Addis Ababa, Ethiopia; 8 Data Use Partnership (DUP), JSI, Addis Ababa, Ethiopia; 9 Minister, Ministry of Health, Addis Ababa, Ethiopia

**Keywords:** HIS infrastructure, Data quality, Data use, Health information system

## Abstract

**Background:**

A strong health information system (HIS) is one of the essential building blocks for a resilient health system. The Ministry of Health (MOH) of Ethiopia is working on different initiatives to strengthen the national HIS. Among these is the Capacity-Building and Mentorship Partnership (CBMP) Programme in collaboration with public universities in Ethiopia since November 2017. This study aims to evaluate the outcomes and share experiences of the country in working with universities to strengthen the national HIS.

**Methods:**

The study employed a mixed-methods approach that included 247 health organizations (health offices and facilities) of CBMP-implementing woredas (districts) and 23 key informant interviews. The programme focused on capacity-building and mentoring facilities and woreda health offices. The status of HIS was measured using a connected woreda checklist before and after the intervention. The checklist consists of items related to HIS infrastructure, data quality and administrative use. The organizations were classified as emerging, candidate or model based on the score. The findings were triangulated with qualitative data collected through key informant interviews.

**Results:**

The results showed that the overall score of the HIS implementation was 46.3 before and 74.2 after implementation of the programme. The proportion of model organizations increased from 1.2% before to 31.8% after the programme implementation. The health system–university partnership has provided an opportunity for higher education institutions to understand the health system and tune their curricula to address real-world challenges. The partnership brought opportunities to conduct and produce local- and national-level evidence to improve the HIS. Weak ownership, poor responsiveness and poor perceptions of the programme were mentioned as major challenges in programme implementation.

**Conclusion:**

The overall HIS has shown substantial progress in CBMP implementation woredas. A number of facilities became models in a short period of time after the implementation of the programme. The health system–university partnership was found to be a promising approach to improve the national HIS and to share the on-the-ground experiences with the university academicians. However, weak ownership and poor responsiveness to feedback were the major challenges identified as needing more attention in future programme implementation.

**Supplementary Information:**

The online version contains supplementary material available at 10.1186/s12961-021-00787-x.

## Background

Health systems consist of all the people and actions whose primary purpose is to improve health [1]. The health system refers to the institutions, people and resources involved in delivering healthcare to individuals. Health systems are one of several determinants of health, and high-performing health systems can improve the health of populations [2]. Health information systems (HIS) are the interaction between people, processes and technology to support operations and management in delivering essential information to improve the quality of healthcare services [3]. The role of HIS is to generate, analyse, use and disseminate data [4]. Having a strong HIS has resulted in benefits such as improving data analytics skills, enhancing collaborative care, increasing cost efficiency and advancing population health management.

A weak HIS is characterized by poor data quality and low information use for evidence-based planning and decision-making. Incorrectly and poorly generated healthcare data create multifaceted problems that can seriously increase the burden of diseases on individuals and the community in general [5]. Making healthcare data available and using it for clinical practice and administrative decision-making is an important neglected step to improving the performance of leaders and practitioners in their decision-making [6]. Healthcare data are being challenged with a number of parameters that directly or indirectly affect their quality, and facts that are being generated are not worthy of trust and application for evidence-based decision-making [7]. Evidence shows that strengthening health workforces and leaders results in better achievement of desired outcomes [8, 9].

Ethiopia has been implementing different initiatives to strengthen the national HIS, including the revision of the national health management information system (HMIS) in 2017, with the development of the information revolution roadmap that helped to guide the implementation of the information revolution. This initiative aimed to maximize the availability, accessibility, quality and use of health information for decision-making processes through the appropriate use of information communication technologies to positively impact the access, quality and equity of healthcare delivery at all levels. The information revolution roadmap also conceptualized the development of a connected woredas strategy, with data-use innovations to achieve the highest standards in data quality and use. This pathway begins with a grading process whereby health facilities are evaluated and scored against a common set of criteria related to HIS infrastructure and capacity, data quality, and administrative and clinical data use. Facilities and woredas that meet the highest standards and are able to access and share data with higher levels through offline mechanisms are recognized as “model facilities” and “model woredas”. Model facilities and woredas that take this one step further by enabling online data access and transmission are recognized as “connected facilities” and “connected woredas”.

Following the development of the information revolution roadmap, efforts have been made to improve data quality and use of information at lower levels of the health system. These efforts have included capacity-building training on data recording and reporting, data analysis, quality assurance, routine data use for decision-making, and troubleshooting. Moreover, health information technology initiatives such as the District Health Information System 2 (DHIS2), electronic medical records, Electronic Community Health Information System (eCHIS) and human resource information systems have been implemented to facilitate information systems and improve the delivery of health services [10].

Though Ethiopia has made remarkable progress in implementing HIS activities in the past few years, a thread of evidence shows that the level of data quality and use of information for decision-making is still not satisfactory [11, 12]. Poor HIS infrastructure (such as lack of access to the Internet, inadequate power supply and insufficient medical record rooms), inadequate support, low staff commitment, poor data analytics skills, high attrition rates among health information technicians, and limited use of e-health applications or digitalization have contributed to poor data quality and low information use [13–15]. The Ministry of Health (MOH) decided to engage higher education institutions to take part in strengthening HIS within their catchment areas. In Ethiopia, 46 public universities were actively operating, with a total of 895,675 students enrolled, as of 2019 [16]. The growing number and distribution of higher education institutions allows them to be exploited as an alternative resource for health system development in the respective catchment areas.

Evidence has shown that engagement of higher education institutions could have a substantial impact in terms of capacity-building, technology transfer, training, collaborative research and internship [17–20]. The close relation between universities and service industries or businesses enables the establishment of contextually meaningful curricula. The linkage also provides the opportunity for universities to contribute to developing their community [21]. The Ethiopian Growth and Transformation Plan (GTP 2010) and higher education institution proclamations also support the responsibility of higher education institutions to forge relationships with industries (Article 26:5), avail knowledge and skill to the community (Article 26.6) and conduct joint research projects with industries (Article 24.4) [22, 23].

Despite encouraging efforts, the engagement of higher education institutions in strengthening the HIS was limited. Hence, the MOH, in collaboration with higher education institutions, introduced a new model known as the Capacity-Building and Mentorship Partnership (CBMP) Programme. This programme envisioned establishing ownership and capacity among higher education institutions to collaborate with local health administrations on investigating gaps and designing and implementing innovative interventions to strengthen the HIS. Therefore, this paper aimed to share the vision, approach and experiences of the CBMP initiative with the global health community and assess its outcomes and the underlying implementation challenges in order to strengthen the programme in the next implementation phase.

## Methods

### Study approach

This study employed a mixed-method approach. For qualitative analysis, a phenomenological approach was used to explore the programme specifically from its inception to its current implementation, as the experts themselves were engaged in the design and implementation of the programme and documented the experiences, implementation challenges, success stories and future prospects, which are used as a base for this study. Additionally, key informant interviews were conducted with key decision-makers and implementers who were involved throughout the programme. This study considered a review of routine reports from implementing universities and national documents in line with the implementation of the CBMP programme. Quantitatively, the status of HIS was evaluated before and after the implementation of the CBMP programme.

### Description of the programme

The CBMP programme was introduced by the MOH in collaboration with a group of universities. The CBMP is an initiative envisioned to establish a partnership between health systems and universities to strengthen the national HIS through different interventions. Six universities, namely Addis Ababa, Haromaya, Hawasa, Jimma, Mekele and the University of Gondar, were selected to implement this partnership programme. The programme included all Ethiopian regional states and city administrations. The partnership was aligned as Addis Ababa University with Addis Ababa City Administration; Haromaya University with the Somali and Harari regions and Dire Dawa city administration; Hawasa University with Sidama and the Southern Nations, Nationalities and Peoples’ (SNNP) region; Jimma University with the Oromia and Gambela regions; Mekele University with the Tigray and Afar regions; and the University of Gondar with the Amhara and Benishangul-Gumuz regions.

The selected universities were to work closely with the consortium universities and the nearby health science colleges in capacity-building and mentorship activities. The intervention targeted a group of officials and data users from the woreda health office, health centres and healthcare providers. The intervention began in November 2017, immediately after baseline assessment. The end-line assessment was conducted after 3 years of implementation, and the programme is still active. With simultaneous effort on data quality, data analysis and data use, the staff at each level of the health system were poised to take advantage of increased available data. Through targeted training paired with mentoring and supportive supervision and training for supervisors, capacity-building efforts aimed to contribute to sustainable improvements in health system capacity.

Components of the activity were engagement with stakeholders, training, mentoring and planning for sustainability (Table 1)*.*

**Table 1:** Interventional package for the CBMP programme

S. no.	Interventional package	Strategy	Frequency
1	Capacity-building	Short-term basic training including data quality and information use, root cause and capstone project, computer maintenance and troubleshooting, data analysis, report writing and bulletin preparation	Tailored and need-based
		Short-term training for pre-service on data quality and information use	Before starting the actual work
		Long-term health informatics undergraduate and postgraduate training	Every year
2	Mentorship and supervision	Mentorship	Every 2 months
		Supervision	Every quarter
		Follow-up	Every month
3	Resource mapping and mobilization	HMIS tools and material mapping and distribution	Need-based
4	Monitoring, evaluation and learning	Stakeholder engagement workshop	Biannual
		Woreda-level performance meeting	Every quarter
		Regional-level meeting	Biannual
		National-level meeting	Biannual
		Evidence generation and research	

### Outcome measurement

The primary outcome of the study was the HIS implementation status, which was measured using an information revolution-connected woreda checklist and scoring system developed by the MOH. This approach analysed health facilities and woreda health offices along three dimensions: HIS capacity and infrastructure, data quality and information use. The checklist was adapted from the PRISM [Performance of Routine Information System Management] assessment tool. To evaluate the HIS performance of the facilities and woreda health offices, a connected woreda checklist and scoring system was used (Additional file 1). The scoring of HIS consists of HIS infrastructure, data quality and administrative data use. The HIS infrastructure domain consists of seven main items that account for 30% of the overall score. The data quality assessment domain has five main items that account for 30%, and administrative data use has 10 main items that account for 40% of the overall score. Health facilities that scored less than 65% of assessment criteria were classified as “emerging”, those scoring between 65% and 90% were classified as “candidate”, and facilities that scored above 90% of assessment criteria were classified as a “model facility”.

### Study population

All health facilities and woreda health offices that implemented the CBMP programme were included in the quantitative study. For the qualitative study, key informants were selected according to key roles in the HIS by virtue of their positions. Decision-makers and experts from the MOH, regional health bureaus, zonal health offices, woreda health offices and selected health facilities working on the CBMP programme were eligible to be included in the key informant interviews. In addition, directors or deputy directors of the CBMP programme under the six implementing universities were included in the key informant interviews. A total of 23 key informants were interviewed.

### Data management and analysis

A data extraction sheet was prepared, and data were extracted based on selected variables of interest. Data were cleaned and analysed using Excel and STATA 14 software. A descriptive analysis was conducted to assess the performance of the implementing health facilities and health offices with regard to data quality, and data use was analysed and reported in the form of percentages. Comparisons between the baseline and current performance of health facilities and woreda health offices were made for the consecutive years of implementation. In addition, trend analysis was conducted to show progress over time in selected programme indicators.

A thematic analysis was done for the qualitative data. Audio data and field notes were transcribed in Amharic and translated to the English language. We coded and categorized all the codes into themes.

## Results

Overall, 247 organizations were involved in the CBMP programme implementation. Of these, 38 were woreda health offices, 35 were hospitals and 169 were health centres. Similarly, 247 organizations (38 woreda health offices, 34 hospitals and 174 health centres) participated in the end-line assessment (Table 2).

**Table 2:** Number of organizations included in the programme

Region	Woreda health office			Hospital			Health centre			Total		
	Baseline	Midline	End-line	Baseline	Midline	End-line	Baseline	Midline	End-line	Baseline	Midline	End-line
Addis Ababa	3	3	3	2	2	2	30	31	31	35	36	36
Afar	2	3	3	1	1	1	11	12	12	15	16	17
Amhara	5	5	5	4	4	4	22	25	25	31	34	34
Benishangul-Gumuz	2	2	2	1	1	1	2	4	4	5	7	7
Dire Dawa	0	0	0	2	2	2	8	8	8	10	10	10
Gambela	2	2	2	2	2	2	4	4	4	8	8	8
Harari	6	4	6	2	2	2	7	7	7	15	13	15
Oromia	5	5	5	6	6	6	28	28	28	39	39	39
Sidama	2	3	3	3	3	3	16	16	16	21	22	22
SNNP	3	3	3	5	5	5	15	15	15	23	23	23
Somali	4	4	2	3	3	1	9	9	5	16	16	8
Tigray	4	4	4	4	5	5	17	19	19	29	28	28
Total	38	38	38	35	36	34	169	178	174	247	252	247

### Health information system

The overall score of the HIS implementation was 46.3 before implementation, 58 at the midline and 74.2 after implementation of the programme. The HIS infrastructure, data quality and administrative data use practice increased along the baseline, midline and end-line assessments. Data quality in particular showed a significant improvement over the course of the implementation (Table 3). This finding is supported by qualitative data.

*"The CBMP programme has played an important role in improving the health data quality and enhancing the capacity of health workers. Data completeness and timeliness improved and sense of ownership towards data quality was improved both at individual and hospital level"*. (Hospital director, aged 31 years)

**Table 3:** Status of HIS among CBMP programme-implementing woredas in Ethiopia

Criteria	Score		
	Baseline	Midline	End-line
HIS infrastructure (scored from 30)	15.4	18.9	22.8
Data quality (scored from 30)	12	19.1	25.4
Administrative data use (scored from 40)	18.9	20	26
Overall HIS status	46.3	58	74.2

### Infrastructure

Regarding the status of HIS-related infrastructure, a total of 242, 252 and 245 organizations were assessed at baseline, midline and end-line, respectively. The highest HIS infrastructure score was 22 in Addis Ababa, and the lowest score of 11 was reported in the Oromia region during the baseline survey. During the end-line assessment, Amhara and Oromia regions achieved a score of 24 and 23, respectively. The Gambela region scored the greatest improvement during the end-line evaluation compared with the baseline, followed by Oromia, whereas SNNP and Benishangul recorded the lowest change (Table 4).

**Table 4:** HIS infrastructure, data quality and administrative data use

Region	HIS infrastructure			Data quality			Administrative data use		
	Baseline	Midline	End-line	Baseline	Midline	End-line	Baseline	Midline	End-line
Addis Ababa	22	26	26	17.6	28.1	28.8	26	32	32
Afar	12	19	22	8.8	22.0.8	25.1	13.4	25	26
Amhara	17	18	24	10.8	22.9	28	16.8	19	32
Benishangul-Gumuz	16	14	19	10	19.1	25.1	14.1	11	23.2
Dire Dawa	15	20	25	9.8	20.9	24.2	23	17	21.2
Gambela	11	17	24	8.9	13.4	24.9	12	12	22
Harari	17	18	21	10.2	16.2	20.8	19	13	18
Oromia	11	17	23	9.7	20.3	25.2	12	18.3	28
Sidama	19	23	24	21.8	23.7	27.1	28	29	32
SNNP	17	21	20	13.7	25.5	24.8	21	29	25
Somali	12	11	20	4.9	12.6	20.9	15	6	19
Tigray	16	23	26	17.7	26.9	29.5	26	29	33
National average	15.4	18.9	22.8	12	19.1	25.4	18.9	20	26

HIS infrastructure score (maximum 30), data quality score (maximum 30) and administrative data use score (maximum 40)

### Data quality

For the data quality assessment, nationwide, a total of 242 woreda health offices, health centres and hospitals were included in the baseline assessment. In the baseline assessment, the level of data quality ranged from 4.9 in the Somali region to 17.6 in the Addis Ababa city administration. All regions except Sidama, Tigray and Addis Ababa scored below half. The overall data quality status score was 12.9 in the baseline assessment. All regions showed improvement, and the end-line assessment indicated that a massive improvement was observed in the majority of the regions. Four regions, namely Addis Ababa, Amhara, Sidama and Tigray, achieved the national target for data quality status, while only two regions, Harari and Somali, scored below 80% in the end-line assessment. The overall data quality status increased from 12.9 to 26.3 (Table 4). This was explained by the intensity of the interventions given, the involvement of the stakeholders and the availability of infrastructures. One of the key informants also reported:

*"Introducing the CBMP programme to the health system played an important role in improving the health data quality and enhancing the capacity of health workers and leaders at different levels of the health system".* (Expert, aged 34 years)

### Administrative data use

The overall administrative data use practice score increased from 18.9 at the baseline to 25.9 at the end-line. Region-wise, except for the Harari region and Dire Dawa city administrations, there has been a change in the administrative data use practice. The improvement was higher specifically in the Addis Ababa, Amhara, Sidama and Tigray regions (Table 4).

### Connected woreda classification

**Fig. 1:**
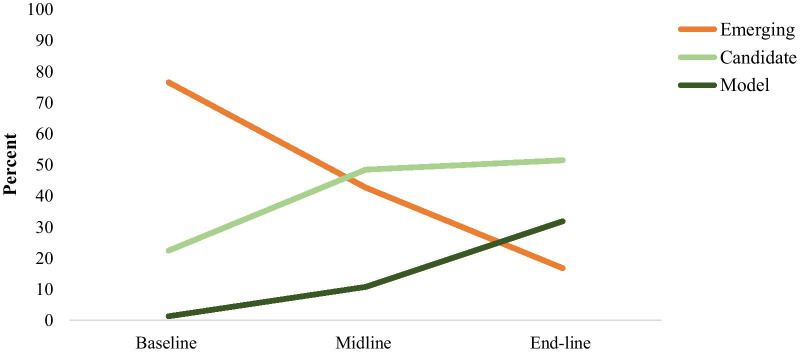
Trend in health information system implementation status

Prior to the execution of the CBMP programme, 76.4% of the organizations were classified as emerging. The proportion of emerging organizations later decreased to 42.6% at the midline and 16.7% after the intervention. The proportion of candidate organizations increased from 22.3% at baseline to 48.4% at the midline and 51.4% after the intervention. Similarly, the model organization increased from 1.2% at baseline to 10.7% at midline and 31.8% after execution of the CBMP programme (Fig. 1).

The baseline assessment showed that all health organizations in Afar, Benishangul-Gumuz, Dire Dawa, Gambela, Harari and Somali were classified as emerging. On the other hand, one (4.4%) of the health organizations in the SNNP and two (9.5%) in the Sidama regions already had model organizations before CBMP implementation. Some regions, including Afar, Dire Dawa, Harari and Somali, had not yet achieved model organizations after the CBMP intervention. After implementation of the CBMP, the Tigray region achieved the highest number, with 19 (67.9%) model organizations, followed by 18 (50%) in Addis Ababa, 10 (45.5%) in Sidama and 15 (44.1%) in the Amhara region (Table 5).

**Table 5:** Number of organizations in emerging, candidate and connected status by region

Status by region	Emerging no. (%)			Candidate no. (%)			Model no. (%)		
	Baseline	Midline	End-line	Baseline	Midline	End-line	Baseline	Midline	End-line
Addis Ababa	13 (37.1)	0 (0)	0 (0)	22 (62.9)	20 (55.6)	18 (50)	0 (0)	16 (44.4)	18 (50)
Afar	14 (100)	5 (31.3)	3 (18.8)	0 (0)	11 (68.8)	13 (81.3)	0 (0)	0 (0)	0 (0)
Amhara	27 (87.1)	14 (41.2)	3 (8.82)	4 (12.9)	20 (58.8)	16 (47.1)	0 (0)	0 (0)	15 (44.1)
Benishangul-Gumuz	5 (100)	6 (85.7)	3 (42.9)	0 (0)	1 (14.3)	1 (14.3)	0 (0)	0 (0)	3 (42.9)
Dire Dawa	10 (100)	7 (70)	3 (33.3)	0 (0)	3 (30)	6 (66.7)	0 (0)	0 (0)	0 (0)
Gambela	8 (100)	8 (100)	3 (37.5)	0 (0)	0 (0)	3 (37.5)	0 (0)	0 (0)	2 (25)
Harari	15 (100)	12 (92.3)	8 (53.3)	0 (0)	1 (7.69)	7 (46.7)	0 (0)	0 (0)	0 (0)
Oromia	37 (94.9)	27 (69.2)	2 (5.1)	2 (5.1)	10 (25.6)	31 (79.5)	0 (0)	2 (5.1)	6 (15.4)
SNNP	15 (65.2)	3 (13)	9 (39.1)	7 (30.4)	18 (78.3)	9 (39.1)	1 (4.4)	2 (8.7)	5 (21.7)
Sidama	11 (52.4)	3 (13.6)	2 (9.1)	8 (38.1)	17 (77.3)	10 (45.5)	2 (9.5)	2 (9.1)	10 (45.5)
Somali	16 (100)	16 (100)	5 (62.5)	0 (0)	0 (0)	3 (37.5)	0 (0)	0 (0)	0 (0)
Tigray	14 (56)	2 (7.1)	0 (0)	11 (44)	21 (75)	9 (32.1)	0 (0)	5 (17.9)	19 (67.9)
Overall	185 (76.4)	103 (42.6)	41 (16.7)	54 (22.3)	122 (48.4)	126 (51.4)	3 (1.2)	27 (10.7)	78 (31.8)

### Health system–university linkage, experiences and lessons

Respondents indicated that the introduction of the CBMP programme has provided an opportunity to understand the real context of the health system. In the key informant interviews, participants reported that the programme offered mutual benefits for higher education institutions and the health industry. The higher education institutions have been given the opportunity to understand the real context of the health system in order to tune their curricula to incorporate contextualized content.

*"CBMP supported strengthening the coordination between the health system and universities and sharing experience and knowledge to improve the health system’s performance."* (Expert, aged 34 years)

*"The programme gave us a chance to understand our health system to adjust our curricula to the real context of our health system".* (University staff, aged 36 years)

The health system–university linkage and partnership has offered an opportunity to develop a national human resources roadmap for HIS in collaboration with the MOH, higher education institutions, regional health bureaus and the Data Use Partnership (DUP). The roadmap was developed for a 10-year period, from 2021 to 2030.

The partnership has allowed different stakeholders to be engaged in assessing human resource demand and existing gaps in the competency of HIS manpower. The curriculum revision and harmonization undertaken involved 10 universities, one health science college, the MOH, the Ministry of Education and regional health bureaus in collaboration with the DUP. The revised curriculum is nationally harmonized and distributed to the universities by the Ministry of Education. Following the initiative, the number of universities that launched health informatics programmes expanded from 3 to 10 during the programme period.

In collaboration with the MOH, local universities have developed a nationally harmonized bachelor’s-level health informatics curriculum for a generic and advanced standing programme. The curriculum was approved by the Ministry of Education and was distributed to all universities throughout Ethiopia. To date, about eight universities have begun offering pre-service health informatics programmes using the newly harmonized curriculum. Other universities are expected to launch programmes in the next academic year. The advanced standing curriculum, which is also approved, allows health information technicians to upgrade their qualification to a bachelor’s level.

A joint team composed of the MOH, University of Gondar and other universities developed eight standardized pre-service training modules for major health informatics courses. This effort ensures that graduates are equipped with the knowledge and skills corresponding to the demand in the health sector. It also responds to the gap in trained HIS professionals in Ethiopia. Work will continue to further improve the pre-service teaching quality within partner universities.

One of the aims of the CBMP is capacity-building of HIS cadres. The CBMP programme has given funding support focusing on research and learning opportunities for doctoral and master’s-level students.

Before the implementation of the CBMP, four universities, namely Addis Ababa University, Mekelle University, Jimma University and University of Gondar, were providing HIS-related master's programmes in Ethiopia, each focusing its coursework on different aspects of HIS. Also, the University of Gondar, Debre Markos University, Metu University and Mekelle University were providing HIS-related bachelor’s degree courses. Before 2017, the four universities which were providing HIS-related courses in a bachelor’s degree had different curriculums. After the implementation of the CBMP, 10 universities, in collaboration with the MOH and Ministry of Education, undertook curriculum harmonization for both bachelor’s degree and advanced health information technician training programmes.

Currently, a total of 10 public health universities and three public health science colleges have a bachelor’s degree programme in health informatics, and a total of 814 students are enrolled in the bachelor’s degree programme. More than 150 bachelor’s degree health informatics professionals have graduated.

Also, before the implementation of the CBMP, scholarships for master's and doctoral degrees typically covered the cost of coursework, but they were not able to support additional academic research activities. The programme provided support for 27 master’s and nine PhD students to conduct operational research on developing and testing interventions to expand data use for evidence-based decision-making at health facilities and woreda health offices for better service delivery and management.

The capacity-building activities are designed to link the training programme to information revolution outputs, targets and sustainability mechanisms. It is believed that building capacity in the areas of monitoring and evaluation, research, HIS and technology, and other related disciplines will contribute to achieving the goals of the information revolution and will ensure complete local ownership and sustainability of its programmes.

The strategy that the universities follow is a “learning by doing” approach, which will ensure the availability of the staff services while they are in education. For example, the MOH and regional health bureau staff admitted to PhD and master of science (MSc) studies through this programme are expected to implement activities prioritized in the connected woreda and CBMP programmes in one of the regions. When implementing the programmes, they are expected to attend seminars in the universities and publish their implementation experience in reputable international journals. According to the human resource regulation, the MOH and regional health bureau staff who will be given this opportunity will have to sign a contract with the MOH regarding their service commitment after graduation.

In general, the implementation of the CBMP programme has increased the number of universities and health science colleges with new HIS training programmes, which ultimately increases the number of students enrolled, leading to more competent graduates to fill the human resource gap.

### Challenges of programme implementation

**Fig. 2:**
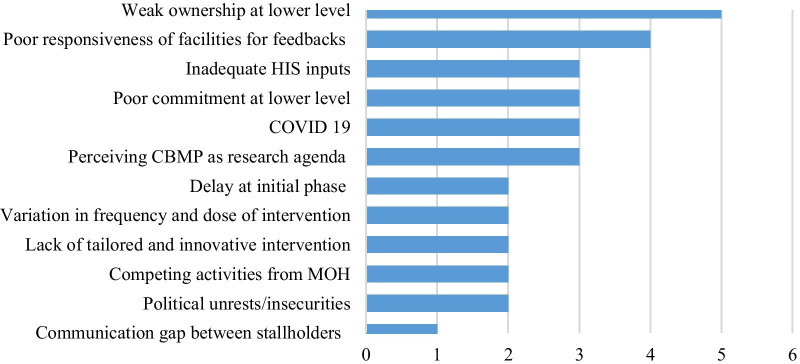
List of challenges in capacity-building and mentorship programme implementation

The study identified major challenges encountered during the implementation of the CBMP programme. Weak ownership at the lower level was reported as the main challenge by the majority of the respondents. Poor facility responsiveness towards the given feedback, perceiving CBMP as a research agenda rather than a partnership, and COVID-19-related difficulties were mentioned as challenges (Fig. 2).

The qualitative findings also identified human resource-related challenges in HIS that included paying less attention to health data, negligence, poor ownership, and weak feedback and response as major challenges. One of the participants noted:

*"We have faced many challenges in implementing HIS activities. Low attention to health data, health information technicians’ negligence due to the job evaluation and grading, weak ownership for the CBMP activities, considering the CBMP activities as a research agenda and tasks of the universities, and weak response to the feedback provided to facilities were some of the challenges for implementing the CBMP".* (Expert, aged 34 years)

## Discussion

This study has shown that the overall status of HIS implementation improved significantly after implementation of the CBMP programme when compared with the status before implementation, as envisioned initially. From the global experience, university–industry linkage brought about substantial results in technology transfer, training, collaborative research and capacity-building [17–19]. Similarly, the partnership with higher education institutions played an enormous role in implementing embedded research, facilitating evidence-based practices and improving the quality of pre-service and in-service training related to HIS. On the other hand, the improvement was also attributable to the attention given by the MOH to the information revolution as one strategic pillar in improving the health system [10].

The study showed improvements in HIS-related infrastructure, although it is still inadequate as compared to the expectation in the national information revolution guideline [24]. Adequate infrastructure is essential to establish a strong HIS at all levels of the health system [25]. Significant variations were also noted among regions in fulfilling resource and infrastructure needs for HIS activities. The highest score for infrastructure was observed in Addis Ababa and Tigray, while the lowest score was in the Benishangul-Gumuz region. This could be because Addis Ababa and Tigray have more facilities located within a short distance for support and follow-up. Additionally, facilities with access to a power supply and the Internet can contribute a larger share to improving HIS performance [26]. Considering the improvement from the baseline to end-line assessment, Oromia showed a large increase, next to the Gambela region. However, Benishangul-Gumuz, SNNP and Addis Ababa showed only slight improvement from the baseline assessment.

The results showed that the overall data quality status was improved at all levels of the health system. It indicated that the improvement in data quality could be due to the interventions that were provided, as most of the gaps identified at baseline were addressed at the health centre, hospital and woreda health office levels. Despite the improvement, data quality was still a major problem. Some of the major gaps, like poor documentation, lack of tally sheets, lack of registers, lack of HMIS guidelines and lack of skill, were identified during the baseline assessment. This is supported by the evidence from the Ethiopian Public Health Institute data quality review report and WHO [9, 27–29]. The assessment helped to design a tailored intervention to improve data quality at the health facility and woreda health office levels. Mentorship, training, supportive supervision, feedback and resource mapping were among the interventions deployed at each level. Capacity-building training on data quality was given for HIS workforces, case team leaders, facility heads and woreda health office heads. Evidence revealed that lack of training is one of the determinant factors for poor data quality status [30]. A joint mentorship and supportive supervision targeting data quality and information use were conducted at the facility and woreda health office levels. The mentorship and supervision were emphasized to solve problems jointly and strengthen two-way communication between supervisors and those being supervised. The improvement in data quality was also explained by the supportive supervision provided by the CBMP programme. Evidence showed that supportive supervision is one of the interventions that can help improve data quality [6, 31]. However, only four regions, namely Addis Ababa, Amhara, Sidama and Tigray, achieved substantial progress in data quality. This implies that data quality is still problematic, and geographical inequalities are observed in data quality.

The study found improvement in administrative data use from baseline to midline and finally to end-line assessment, which was attributable to the CBMP programme, though the overall data use level did not meet the expectation envisioned in the information revolution [10]. The progress made by the CBMP programme results from the continuous capacity-building and mentorship activities carried out in an integrated manner by the health system and affiliated universities. Global evidence has also shown that low-resource settings often have limited use of local data for health system planning and decision-making, and support measures are needed, including technical and capacity-building programmes [32]. Other evidence also indicates that capacity-building interventions are an essential component of a package of HIS-strengthening interventions to improve sustainable demand for and use of data in decision-making at all levels of the health system [33].

The study also revealed that there has been inconsistent performance across regions and health facilities over time. The reasons for the inconsistency may be related to the differences in the frequency and dose in implementing the intervention package. National documents have also reported that achieving cultural transformation in data use is the most challenging part of the information revolution agenda, as it requires strong coordination among different stakeholders at all levels [10, 34]. Utilization of routine data for data-informed decision-making has remained a challenge in Ethiopia [27, 35]. This indicates that integrated efforts like the CBMP programme are required, with tailored interventions for different contexts to achieve cultural transformation in information use at the national level.

In general, the connected woreda status showed a steady improvement from baseline to midline and end-line assessment attributable to the CBMP intervention. However, no single woreda achieved connected status. This implies that online data access and transmission remain challenging, and further efforts are needed to connect all woreda facilities digitally. The connected woreda plan considers the establishment of a widespread culture of using health information for decisions at all levels. However, the process requires reliable electricity and connectivity, digital literacy, standard processes and training for health workers to fully implement digital tools such as electronic patient registry, DHIS2 and eCHIS technologies [36].

The CBMP partnership has also had a substantial impact on pre-service education, as the number of universities and colleges with health informatics training increased from 4 to 13 within 3 years of the partnership, which resulted in the addition of hundreds of professionals that can play a key role in a sustainable and strong national HIS in Ethiopia.

As a limitation, this study focused on administrative data use. We did not address clinical data use practices, so the results may not show the full picture of data use at all levels of the health system.

## Conclusion

The overall HIS has shown substantial progress in capacity-building and mentorship programme implementation in woredas. A number of facilities became models in a short period of time after the implementation of the programme. The health system–university partnership was found to be a promising approach to improve the national HIS and to share the on-the-ground experiences with the university academicians. However, weak ownership and poor responsiveness to feedback were identified as major challenges that need more attention in future programme implementation.

## Supplementary Information

**Additional file 1**. Connected Woreda checklist and point allocations.

## Data Availability

Data will be available upon reasonable request from the corresponding author.

## Abbreviations

CBMP: Capacity-Building and Mentorship Partnership
DHIS2: District Health Information System 2
DUP: Data Use Partnership
HIS: Health information system
MOH: Ministry of Health
MR: Medical record
SNNP: Southern Nations, Nationalities and Peoples
