# Supplementary material for: Strengthening the national health information system through a capacity-building and mentorship partnership (CBMP) programme: a health system and university partnership initiative in Ethiopia
Source: Health Res Policy Syst. 2021 Dec 9;19:141. doi: 10.1186/s12961-021-00787-x (PMC8656449; doi:10.1186/s12961-021-00787-x)
Supplement: Supplementary file 1 — Additional file 1. Connected Woreda checklist and point allocations. [file 12961_2021_787_MOESM1_ESM.docx]

## **ANNEX: CONNECTED WOREDA CHECKLIST AND POINT ALLOCATIONS**

## **Structure and implementation of HIS (30%)**

| SN | Indicators | Possible Points | Points Given |
| --- | --- | --- | --- |
| 1 | **The woreda health office has put in place the inputs needed to strengthen HIS** | **5** |  |
|  | The woreda health office has a well-organized monitoring and evaluation/planning unit   - Dedicated office/desk for HMIS staff – 1 point - A computer dedicated for DHIS 2 is in place and it is currently functioning – 1 point - The M&E/planning unit is fully staffed as per the standard (The Woreda Health Office has assigned personnel dedicated for the implementation of HIS) – 1 point   ***Define staffing standard for a Woreda Health Office M&E/planning unit*** | 3 |  |
|  | At least four manuals which facilitate the implementation of HIS are in place within the Woreda Health Office   - HMIS procedure/data recording and reporting – 0.5 point - HMIS Indicator Reference Guide – 0.5 point - HMIS disease classification (NCOD) – 0.5 point - Data quality and use – 0.5 point | 2 |  |
| 2 | The Woreda Health Office has allocated funds for its health facilities for strengthening implementation of HIS   - If the budget for HIS is greater or equal to 15% of the overall health budget – 4 points - If the budget for HIS is 10-15% of the overall health budget – 3 points - If the budget for HIS is 5-10% of the overall health budget - 2 points - If the budget for HIS is less than or equal to 5% of the overall health budget – 1 point - If there is no budget allocated for the implementation of HIS – 0 points | **4** |  |
| 3 | **The Woreda Health Office (WoHO) has a system for timely supportive supervision with fixed schedule** | **6** |  |
|  | The Woreda Health Office conducted supportive supervision in the past six months as per the national standard   - The WoHO conducted HIS supportive supervision for all the health facilities and health posts under the Woreda Health Office as per the standard - 2 points - The WoHO conducted HIS supportive supervision to some health facilities and health posts under the Woreda Health Office as per the standard – 1 point - The WoHO did not conduct HIS supportive supervision to any of the health facilities and health posts under the Woreda Health Office as per the standard – 0 point | 2 |  |
|  | Supportive supervision is done using a checklist | 1 |  |
|  | Written supportive supervision report/feedback provided to the health facilities   - Written supervision feedback is provided to all the facilities supervised – 2 points - Written supervision feedback is provided to some facilities – 1 point - No supervision feedback was provided after supervision – 0 point | 2 |  |
|  | Action plan prepared during the supportive supervision to facilitate follow-up | 1 |  |
| 4 | **Mentoring conducted to strengthen the HIS (in the past six months)** | **4** |  |
|  | The Woreda Health Office conducted mentoring in the past six months   - All health facilities have received mentoring – 2 points - Some health facilities have received mentoring – 1 point - No mentoring was done at all – 0 point | 2 |  |
|  | The Woreda Health Office conducted mentoring in the past three months based on the national standard   - Mentoring was conducted base on the national standard (mentoring conducted as per the schedule, report produced, and feedback provided) - 2 points - Mentoring was done but not as per the national standard (didn’t follow the mentoring schedule, no report produced, and/or no feedback provided) - 1points - No mentoring was done at all - 0 point | 2 |  |
| 5 | **The health administrative unit has built the capacity of its personnel on health information management, use, monitoring and evaluation**   - Capacity needs was addressed to all staff in the woreda based on capacity gap assessment – 4 points - Capacity needs was addressed to some staff in the woreda based on capacity gap assessment – 3 points - Capacity needs was addressed to all staff even though capacity gap assessment was not done – 2 points - Capacity needs was addressed to some staff even though capacity gap assessment was not done – 1 point - Capacity needs was not addressed at all – 0 points | **4** |  |
| 6 | **A partners forum is in place**   - Joint partners discussion forum was held two times in the past 6 months – 4 points - Joint partners discussion forum was held once in the past 6 months – 3 point - No discussion forum was held in the past 12 months – 0 points | **4** |  |
| 7 | **The Woreda Health Office has implemented eHIS** | **3** |  |
|  | - The Woreda Health Office has implemented DHIS 2 | 1 |  |
|  | - The health administrative unit has implemented eHRIS | 1 |  |
|  | - The health administrative unit has implemented eLMIS | 1 |  |

## **Data Quality (30%)**

| SN | Indicators | Possible Points | Points Given |
| --- | --- | --- | --- |
| 1 | **In the last six months, the woreda has conducted RDQA**   - Conducted RDQA in all the health facilities twice in the last 6 months– 7 points - Conducted RDQA in all the health facilities once in the last 6 months– 6 points - Conducted RDQA in some of the health facilities twice in the last 6 months – 5 points - Conducted RDQA in some of the health facilities once in the last 6 months – 3 points - RDQA has not been conducted in any of the health facilities – 0 point | **7** |  |
| 2 | **In the last six months, the woreda has met the data accuracy target**   - All the assessed indicators scored data verification factor between 0.9 and 1.1 - 5 points - Half or more of the assessed indicators scored data verification factor between 0.9 and 1.1 – 3 points - Less than half of the assessed indicators scored data verification factor between 0.9 and 1.1 - 0 points   *Note: The indicator selection should be based on the national guideline (7-9 indicators)* | **5** |  |
| 3 | **Data accuracy improvement action plan was prepared based on data verification findings in order to address gaps** | **2** |  |
| 4 | **In the last six months, Woreda Health Office and health facilities under it have produced completed reports** | **8** |  |
|  | The woreda Health Office keeps a log book/uses electronic system that helps to track reporting completeness | 1 |  |
|  | Reporting completeness is 90% and above   - 90% or greater of the expected reports have been submitted to the Woreda Health Office in the previous six months – 4 points - Below 90% of the expected reports have been submitted to the Woreda Health Office in the previous six months – 0 points | 4 |  |
|  | Content completeness   - The ’content completeness’ of Woreda Health Office reports already submitted to the next health administrative level were greater or equal to 90% for all the reports – 3 points - The ‘content completeness’ of Woreda Health Office reports already submitted to the next health administrative level was less than 90% only one times - 2 point - The ‘content completeness’ of Woreda Health Office reports already submitted to the next health administrative level was less than 90% more than one times – 0 points | 3 |  |
| 3 | **Reports in the last six months are submitted in a timely manner** | **8** |  |
|  | The Woreda Health Office keeps logbook/uses electronic system that helps to track report timeliness | 1 |  |
|  | Reports are submitted according to the national reporting schedule   - All Woreda Health Office reports in the last six months were submitted to the next level according to the national reporting schedule – 4 points - All Woreda Health Office reports in the last six months were submitted to the next level according to the national reporting schedule in five instances – 3 point - All Woreda Health Office reports in the last six months were submitted to the next level according to the national reporting schedule in four instances – 2 point - All Woreda Health Office reports in the last three months were submitted according to the next level national reporting schedule in three or fewer instances – 0 points | 4 |  |
|  | The Wored Health Office has received reports from its health facilities according to the national schedule   - 90% or greater of expected reports from health facilities in the last six months are received on time according to the national schedule – 3 point - Between 80%-89% of expected reports from health facilities in the last six months are received on time according to the national schedule – 2 point - Less than 80% of expected reports from health facilities in the last six months are received on time according to the national schedule – 0 point | 3 |  |

## **Administrative Data Use (40%)**

| SN | Indicators | Possible Points | Points Given |
| --- | --- | --- | --- |
| **1** | **Performance management team(PMT) is in place and established according to national standard**   - PMT is in place and the members are put together based on the national standard – 2 points - PMT is in place but the members are not put together based on the national standard – 1 point - PMT is not established at all – 0 points   ***Define the membership as per the national standards*** | **2** |  |
| **2** | **PMT is convening on monthly basis**   - PMT has met for six or more times in the last six months - 2 points - PMT has met for five times in the last six months – 1.5 points - PMT has met four or less times in the last six months – 1 point - PMT has not met in the last six months – 0 points | **2** |  |
| **3** | **PMT is chaired by the head of the Woreda Health Office as per the national standard**   - All the PMT meetings in the last six months were chaired by the head of the WoHO – 1 point - At least three PMT meetings in the last six months were chaired by the head of the WoHO – 0.5 point - Less than three of the PMT meetings in the last six months were chaired by the head – 0 point | **1** |  |
| **4** | **PMT is reviewing key performance indicators** | **14** |  |
|  | The health facility is tracking key quality and equity indicators from the transformation plan   - Health quality and equity indicators are included in the list of indicators being tracked -2 points - Either quality or equity indicators are included in the list of indicators being tracked -1 points - There are no quality or equity indicators in the list of indicators being tracked – 0 points   ***For equity: there is documented information that shows comparison of key performance indicators on urban/rural, age and sex disaggregation***  ***There is documented evidence that shows tracking key quality indicators (define these indicators)*** | 2 |  |
|  | Plan versus achievement based on the key indicators   - There is documented information that shows comparison was made between what is planned and what is achieved on the key indicators six times in the last 6 months- 3 points - There is documented information that shows comparison was made between what is planned and what is achieved on the key indicators five times in the last 6 months- 2 points - There is documented information that shows comparison was made between what is planned and what is achieved on the key indicators four or less times in the last 6 months- 1 point - There is no documented information that shows comparison is made between what is planned and achieved based on the key indicators - 0 points | 3 |  |
|  | Performance gaps are identified by comparing achievement against target | 1 |  |
|  | Root cause analysis is done for low performing key indicators   - Root cause is identified for all low performing key indicators – 2 points - Root cause is identified for only some low performing indicators – 1 points - Root cause is not identified for all the low performing indicators – 0 points | 2 |  |
|  | Action plan is prepared for the identified priority problems/challenges   - Action plan (with roles and responsibilities, resources and timeline) is prepared for all the identified priority problems/challenges – 2 points - Action plan is prepared for some of the identified priority problems – 1 points - Action plan is not prepared at all – 0 points | 2 |  |
|  | The action plan is being implemented   - There is documented evidence for actions taken – 2 points - No action is taken– 0 points | 2 |  |
|  | PMT action plan/meeting minutes were circulated to case teams   - PMT action plan/meeting minutes were circulated to case teams six times in the last six months – 2 points - PMT action plan/meeting minutes were circulated to case teams five times in the last five months – 1.5 points - PMT action plan/meeting minutes were circulated to case teams four or less time in the last six months – 1 points - PMT action plan/meeting minutes were not circulated to the case teams at any point in the last three months - 0 points | 2 |  |
| **5** | **Written feedback was given to lower level supervisory unit on strengths and weaknesses based on the analysis of information collected**   - The Woreda Health Office provided written feedback to all lower level supervisory units two times in the last six months – 6 points - The Woreda Health Office provided written feedback to all lower level supervisory units once in the last six months – 5 points - The Woreda Health Office provided written feedback to only some lower level supervisory units two times within the last six months – 4 points - The Woreda Health Office provided written feedback to only some lower level supervisory units only once in the last six months – 3 points - The Woreda Health Office has not provided written feedback to lower level supervisory units- 0 points | **6** |  |

| 6 | The Woreda Health Office has presented or disseminated at least one assessment findings in the last six months   - The Woreda Health Office has conducted and disseminated at least one assessment findings – 3 points - The Woreda Health Office has conducted at least one assessment findings but unable to disseminate - 2 points - The Woreda Health Office has not presented or disseminated any assessment findings - 0 points   *The assessment could include client satisfaction survey, waiting time, case studies, case report, equity survey, etc.* | 3 |  |
| --- | --- | --- | --- |
| 7 | **Every case team has a program performance monitoring chart**   - All case teams have displayed a program performance monitoring chart – 3 points - Only some of the case teams have displayed a program performance monitoring chart – 2 points - Only the HMIS unit/case team has displayed a performance monitoring chart – 1 point - None of the case teams have a program performance monitoring chart – 0 points   ***Provide standard list of performance monitoring chart*** | **3** |  |
| 8 | **The Woreda Health Office has displayed information in the form of table, chart, etc. based on selected indicators in the office compound and in the community**   - Information is displayed in the Woreda Health Office compound and other community locations – 3 points - Information is displayed only in the Woreda Health Office compound or in community locations only - 2 points - No information was displayed either in the Woreda Health Office compound or other community locations – 0 points | **3** |  |
| 9 | **Information dissemination materials such as a brochure or newsletter that shows the woreda health performance is printed and disseminated to the general public**   - A brochure or newsletter or other printed materials that shows the woreda performance was printed and disseminated twice in the last 6 months - 4 points - A brochure or newsletter or other printed materials shows the woreda performance was printed and disseminated once in the last six months – 3 points - A brochure or newsletter or other printed materials that shows the woreda performance was printed but not disseminated in the last six months – 2 point - No brochure or newsletter is printed and disseminated in the last twelve months – 0 points | **4** |  |
| 10 | **The Woreda Health Office held performance review meeting with stakeholders**   - The WoHO held review meeting twice in the last six months – 1 point - The WoHO held review meeting once in the last six months – 0.5 point - The WoHO did not held performance review meeting in the last six months – 0 point | **1** |  |
